# Do Organizational Health Climates and Leader Health Mindsets Enhance Employees’ Work Engagement and Job Crafting Amid the Pandemic?

**DOI:** 10.3390/ijerph182212123

**Published:** 2021-11-18

**Authors:** Yuhyung Shin, Won-Moo Hur

**Affiliations:** 1 School of Business, Hanyang University, 17 Haengdang-dong, Seongdong-gu, Seoul 133-791, Korea; yuhyung@hanyang.ac.kr; 2 College of Business Administration, Inha University, 100 Inha-ro, Michuhol-gu, Incheon 22212, Korea

**Keywords:** COVID-19, organizational health climate, leader health mindset, work engagement, job crafting

## Abstract

Although the COVID-19 pandemic has resulted in many health- and stress-related symptoms among employees, surprisingly few studies have assessed the effect of a health-promoting organizational climate or leadership on employee work outcomes. To fill this gap, our research proposed and tested a moderated mediation model involving perceived organizational health climate (POHC), leader health mindset (LHM), work engagement, and job crafting. Our propositions were tested using two-wave data collected from 301 South Korean employees. As predicted, POHC was positively related to employees’ job crafting, and this relationship was mediated by work engagement. Moreover, the positive relationship between POHC and work engagement and the indirect effect of POHC on job crafting through work engagement were more pronounced when LHM was high than when it was low. These findings support the job demands–resources model and social exchange theory and have implications for helping employees maintain their work attitudes and behavior in times of crisis.

## 1. Introduction

The COVID-19 pandemic has drastically altered employees’ working situations [1]. Owing to concerns about potential virus transmission, perceived threats to job security, and feelings of isolation caused by social distancing and telework, employees have been exposed to an increasing number of job stressors throughout the pandemic [2]. In the current crisis situation, motivating employees to remain engaged and productive and eliminating obstacles that may harm their occupational health are dilemmas that organizations must resolve. While COVID-19 research highlights the role of positive or supportive leadership and organizational climates as a buffer against stress experienced by employees [3], surprisingly few studies have assessed the effects of a health-promoting climate or leadership style on work outcomes in the context of the pandemic. Thus, whether health-promoting climates are conducive to employee work outcomes during the COVID-19 pandemic and under what conditions this positive effect is strengthened constitute timely and necessary research agendas. To answer these questions, our research aims to examine the effect of perceived organizational health climates (POHCs) on employee work outcomes and explore a boundary condition that boosts the positive effect of POHC in the pandemic context.

POHC refers to perceptions of organizational policies and practices that promote the physical and psychological health of employees [4,5]. We assume that POHC has become more important amid the pandemic, where employees are faced with increasing threats to their physical and psychological health. It is speculated that health-promoting organizational policies and procedures protect employees from stressors associated with the pandemic and enable employees to remain engaged and proactive at work. In this study, we focus on work engagement and job crafting as crucial outcomes. Work engagement refers to a state of feeling vigorous, dedicated, and absorbed in one’s job [6]. Job crafting is a form of proactive behavior that modifies one’s work content and work-related cognitions so that one’s job is more motivating and meaningful [7]. We propose job crafting as the dependent variable and work engagement as a mediator linking POHC and job crafting. As concerns about potential virus transmission, social distancing, and job security prevent employees from feeling engaged at work [2], whether employees maintain their work engagement in a high POHC is an important research topic in the pandemic context. We propose that POHC enhances employees’ work engagement, which, in turn, positively affects their job crafting. We contend that job crafting is a crucial work behavior in a pandemic situation, which is characterized by a high level of uncertainty. The COVID-19 pandemic is an ongoing crisis in many countries, and it is difficult to forecast when it will end. Under this environmental uncertainty, uncertainty-reducing behavior such as job crafting (e.g., seeking information or feedback necessary for task performance) can help employees to effectively react to and deal with environmental changes [8]. Moreover, given that it is difficult for managers to closely monitor and supervise employee behaviors due to the social distancing and teleworking setups during the pandemic, employees’ discretionary behavior that autonomously crafts their own work is deemed desirable and effective [9,10,11,12,13]. As such, the first objective of our research is to propose and test the mediating relationship between POHC, work engagement, and job crafting during the COVID-19 pandemic.

Our research also aims to explore a boundary condition that strengthens the positive relationship between POHC, work engagement, and job crafting. We identify the leader health mindset (LHM; i.e., awareness of employee health issues and concerns [4]) as a key moderator that interacts with POHC. Organizational research has shown that organizational culture or climate and leadership style interact to affect employee outcomes [14,15]. Drawing on these findings, a study of organizational health is incomplete without jointly considering the roles of organizations and leaders in promoting employee health and work outcomes. Nonetheless, prior work conducted in the pandemic context has not uncovered the interaction effect between POHC and LHM. While Kaluza et al. [4] demonstrated that POHC has a cascading effect on employee outcomes by enhancing the leader health mindset, this finding has not been validated in the context of the pandemic. Klebe et al. [1], in their study of the role of health-oriented leadership amid the pandemic, reported that health-oriented leadership is particularly important for followers facing a crisis and has a positive relationship with follower performance. However, this study did not examine POHC as an antecedent to follower outcomes. The organizational health literature asserts that POHC and leader support for health promotion make unique contributions to employee work outcomes [16,17]. Whereas POHC reflects organization-wide support and initiatives to promote employee health, health-promoting leadership or LHM pertain to supervisors’ awareness of and sensitivity toward employee health issues [4]. Drawing on this contention, we propose LHM as a moderator that strengthens the positive effect of POHC on work outcomes. Thus, the second objective of our research is to investigate the moderating effect of LHM on the POHC–work engagement relationship and the POHC–work engagement–job crafting relationship during the COVID-19 pandemic.

Our research makes several contributions to the literature on organizational health and job crafting. First, organizational health research has generally examined the roles of POHC and health-promoting leadership separately. As noted earlier, only a handful of studies have assessed the interaction effect between these two variables. By proposing LHM as a boundary condition that facilitates the positive effect of POHC, our research provides a more elaborate picture of how POHC and LHM interplay in a crisis situation such as the COVID-19 pandemic. Second, despite the importance of POHC and health-promoting leadership in times of crisis, only one study has tested the effect of health-promoting leadership on the outcomes of employees as affected by the COVID-19 pandemic [1]. Our research fills this void by elucidating the interaction effect of POHC and LHM on employee work outcomes during the pandemic. Furthermore, unveiling the unknown roles of POHC and LHM during the pandemic offers practical knowledge to help employees cope with work stressors in such an unprecedented crisis. Finally, our research advances the job crafting literature by treating health-promoting climate as an antecedent to job crafting. Distinct from prior research that posits diversity climate [18] and climate for innovation [19,20] as a work context promoting job crafting, our study is the first to identify POHC as a predictor of job crafting.

## 2. Theoretical Background and Hypotheses

### 2.1. Theoretical Foundations

As stated above, our research aims to examine the mediating relationship between POHC, work engagement, and job crafting and the moderating effect of LHM on this relationship. We adopt the job demands–resources (JD–R) model and social exchange theory (SET) as theoretical underpinnings for our hypotheses. The main tenet of the JD–R model is that job features are categorized into job “demands” and “resources” depending on whether they are conducive or detrimental to employees’ goal attainment and personal development [21,22]. “Job demands” are the aspects of a job that incur stress among employees and hinder their goal attainment and personal development; examples include job insecurity, workplace conflict, work overload, and role ambiguity. “Job resources” are the aspects of a job that perform functions opposite to those of job demands [22]. Autonomy, feedback, opportunities for growth and development, and social support are specific examples of job resources.

The JD–R model theorizes that job resources promote employees’ work behavior and performance by enhancing their work engagement, whereas job demands undermine their work outcomes through burnout—specifically, job resources have been found to positively predict job crafting through work engagement [23]. As a proactive behavior that alters the content and scope of one’s job and enriches one’s relationships with others in the workplace, job crafting requires a substantial amount of resources. Job resources not only alleviate strain but also buffer the deleterious effects of job demands on strain [21,24]. According to this perspective, POHC acts as a job resource because it reflects top management’s support for workplace health promotion [25]. Likewise, LHM—by providing support for employee health issues—functions as a job resource for employees experiencing the COVID-19 pandemic [26]. Therefore, it is presumed that POHC and LHM provide those employees under such a crisis with resources that can buffer them against health-related stress. Consequently, employees are likely to feel engaged in their work and can proactively craft their jobs.

The relationship between POHC, LHM, work engagement, and job crafting can also be explained by SET, which assumes that an employee and their employer (or leader) engage in a social exchange relationship with each other. In other words, when employees receive resources and are treated well by their organization and leader, they reciprocate with desirable work attitudes and behaviors in return for such positive treatment [27]. If employees feel that they have a high-quality relationship with the organization, they tend to behave in a manner that benefits the organization [5]. In particular, when employees perceive that their organizations and leaders are concerned about their health and take actions to promote health, they tend to display more positive work attitudes (i.e., work engagement) and behave more proactively (i.e., job crafting). Grounded in the JD–R model and SET, we propose a positive relationship between POHC, work engagement, and job crafting and the moderating effect of LHM on this relationship. The proposed relationships are explained in more detail in the following sections.

### 2.2. Relationship between POHC, Work Engagement, and Job Crafting

Organizational climate is defined as a subjective representation of current practices, policies, and procedures in an organization [28]. POHC is a specific facet of organizational climate that represents employees’ perceptions that their organization actively cares for and supports the physical and psychological well-being of employees [5]. While Zweber et al. [5] conceptualized POHC as a multidimensional construct that encompasses health-related support from the organization, workgroup, and supervisors, we focus on the facet entailing support from the organization, as it reflects the overall organizational policies, practices, and procedures for promoting employee health. Furthermore, as we focus on supervisors’ health mindset in our study, the supervisory aspect of POHC might overlap with this construct. Therefore, we confine the conceptualization of POHC to the organizational dimension. Organizational health research has indicated that health climate has a positive relationship with employee performance, job satisfaction, and health and a negative relationship with job stress, burnout, and turnover intentions [5]. Although the relationship between POHC and job crafting has never been empirically tested, we propose that POHC will be positively associated with job crafting during the pandemic.

Job crafting can take three forms: (1) modifying the number and scope of tasks (i.e., task crafting); (2) altering the amount and intensity of interactions with others in the workplace (i.e., relational crafting); and (3) cognitively reframing the meaning and importance of the job (i.e., cognitive crafting) [7]. In turbulent times such as the COVID-19 pandemic, employees need to engage in uncertainty-reducing behavior (e.g., asking for feedback on their performance, adjusting the content and scope of their tasks to environmental demands, altering the nature and boundary of work relationships according to the changes in working conditions, and ensuring that their job is emotionally less tense [8]). To engage in these job crafting behaviors, employees need physical and mental resources. We claim that POHC plays a pivotal role in a pandemic situation where employees are faced with increasing stressors (e.g., concerns about COVID-19 infection, perceptions of job insecurity, and feelings of isolation). Organizational interventions to promote employee health (e.g., organizing social and interpersonal health-promoting activities, monitoring employee health symptoms, providing psychological counseling, and using flexible scheduling arrangements) offer the psychological resources necessary for coping with COVID-19-related stressors [29]. These interventions further enable employees to maintain their physical and psychological health even in the presence of the pandemic, which provides the physical and mental resources necessary for changing the number and scope of tasks (i.e., task crafting) and the number and intensity of interactions with others (i.e., relational crafting). As such, in organizations with a high POHC, employees actively craft their jobs by mobilizing their resources to core job functions and adjusting their work and relational boundaries to the volatile changes in the environment. Moreover, from a JD–R perspective, a high level of POHC relieves job demands (e.g., COVID-19-related stressors) experienced by employees, causing them to view their job in a more positive way. In this regard, POHC also facilitates cognitive crafting.

The positive relationship between POHC and job crafting is also supported by SET, which claims that when employees receive resources from the organization, they invest these resources in improving their performance [27,30]. Job crafting researchers position job crafting as a proactive behavior that improves work performance [23]. Thus, when employees perceive that their organization expends resources in health-promoting activities during the pandemic, they likely reciprocate with proactive behavior that improves their performance and working conditions. Consequently, employees of organizations with a high health climate should engage in more job crafting amid the pandemic than those with a low health climate. Hence, the following hypothesis is proposed:

**Hypothesis** **1** **(H1).** *POHC is positively related to employees’ job crafting during the COVID-19 pandemic.*

We further predict that work engagement is a primary mediator of the relationship between POHC and job crafting. This proposition is grounded in the JD–R model, which holds that job resources constitute a motivational path that positively affects work behavior and performance through work engagement [21,22]. According to this framework, job resources—by facilitating goal attainment and employee growth and development—propel employees to remain motivated and engaged even under the threats of the pandemic. In a pandemic situation, characterized by increasing job insecurity and interpersonal stressors [2], employees have difficulty concentrating on their tasks. Health-promoting organizational practices provide organizational resources that help their employees deal with the environmental uncertainty and maintain their work motivation and engagement during the pandemic. Engaged employees, in turn, become capable of effectively allocating resources and coordinating task-related activities [23,31]—all of which are the key components of job crafting. Moreover, engaged employees are eager to improve their current job features and seek more job characteristics amid the pandemic [32], which can be realized through job crafting. Applying this logic to organizational health, a high level of POHC encompasses improving employees’ working conditions so that they are conducive to both physical and psychological health. These improved working conditions serve as resources that help employees cope with the psychological symptoms caused by the pandemic and remain engaged at work, thereby becoming more adaptive to the environment [33] and taking initiative in the workplace [34]. Consequently, engaged employees utilize their resources to proactively modify their work and relational boundaries (i.e., task and relational crafting) according to environmental demands. In addition, as work engagement entails positive emotions [33], engaged employees likely see their current job conditions in a positive light, thereby being capable of cognitively crafting the job in the pandemic context.

The mediating relationship between POHC, work engagement, and job crafting can also be explained by SET. When employees perceive that their organization cares about employees’ well-being and take actions to alleviate their COVID-19-related stress and improve working conditions, they display greater identification with and commitment to the organization in return for the positive treatment [4]. Thus, they become more dedicated to and absorbed by work and engage in proactive behavior to craft their jobs even under the threats of the pandemic. This line of reasoning leads to the following mediation hypothesis:

**Hypothesis** **2** **(H2).** *Employees’ work engagement mediates the positive relationship between POHC and job crafting during the COVID-19 pandemic.*

### 2.3. Moderation of LHM

While we have proposed the existence of positive ties between POHC, work engagement, and job crafting, conditions can exist that further improve this relationship. Drawing on the JD–R proposition that organizational and social resources synergistically interact to promote employees’ work engagement, we claim that the effect of POHC (i.e., organizational resource) can be boosted when leaders (i.e., social resource) support this kind of climate. Prior research has shown that health-promoting leader behavior predicts employee well-being more strongly than other leader behaviors [35,36]. Consistent with these findings, we argue that the mindset of supervisors who interact closely with employees functions as a key social resource strengthening the positive impact of POHC on employee outcomes. A health mindset is known as a key antecedent that directly affects supervisors’ health-promoting behavior. The literature on health leadership suggests that health awareness is a key component of health-promoting leadership [26,35]. Empirical findings indicate a positive relationship between health-promoting leadership and a variety of work outcomes (e.g., well-being, physical health, job performance, and satisfaction) [4,26,35,37,38,39,40]. Based on these findings, we anticipate that LHM—a core component of health-promoting leadership—will serve as a condition that amplifies the positive effect of POHC on work outcomes during the pandemic.

Supervisors with a strong health mindset pay attention to employees’ health and stress symptoms [40]. As they are highly aware of employees’ health issues and easily notice whether employees are overworked or exhausted in a highly stressful situation such as the COVID-19 pandemic, they can take necessary actions when such health issues arise [4]. Thus, supervisors with a strong health mindset are likely to fuel the motivational effect of POHC in the context of the pandemic. When employees work in an organization with a high POHC and interact with supervisors with a strong health mindset, they likely exhibit high levels of work engagement and job crafting even in the presence of an imminent environmental threat such as the pandemic. Employees who perceive their supervisors as health-promoting evaluate their job characteristics more positively in a stressful situation [35], which is positively associated with cognitive crafting. Conversely, supervisors with a weak health mindset do not notice signals that employees have reached their health limits during the pandemic [4]. In this case, although the organization implements policies and practices that maintain employee health, employees may not feel engaged at work. As they lack resources for task activities, employees feel less inclined to exert extra effort in conducting their work, resulting in decreased job crafting.

From a JD–R perspective, leader behavior or mindsets serve as a key social job resource for employees who are exposed to work stressors. If supervisors are attentive and sensitive toward employees’ health issues and signals that occurs during the pandemic, employees tend to feel that their supervisors display emotions of care and concern for them. Such a social resource guards employees against strain resulting from the pandemic, making them engaged and proactive at work. However, a low level of LHM is expected to nullify the positive effects of POHC during the pandemic. A high level of POHC and a low level of LHM give employees conflicting signals (i.e., “my organization cares about my well-being during the pandemic, but my supervisor does not”), which causes stress for employees. Consequently, employees are likely to have difficulty concentrating on work, exhibiting decreased work engagement and job crafting.

According to SET, employees reciprocate treatment received from the organization and that received from the supervisor. If the supervisor is genuinely concerned about the physical and psychological health of employees facing the pandemic, employees perceive that they are trusted and respected by the supervisor, thereby forming a high-quality relationship with the supervisor [41]. In particular, given that employees experience many environmental and work stressors during the pandemic, support and concern from the supervisor are highly appreciated by them, which leads to a return of desirable work attitudes and behavior. As such, LHM improves supervisor–employee relationships in the pandemic context by facilitating communication and building trust [42]. In addition, it creates positive emotions among employees and fuels them to expend sustained resources for their work. As a result, a high level of LHM boosts the positive effect of POHC on job crafting through work engagement during the pandemic. Contrastingly, when the supervisor shows little concern for employee health, employees cannot develop a high-quality relationship with the supervisor and, therefore, reduce their work efforts and commitment, although they perceive that their organization treats them well. Consequently, a low level of LHM should attenuate the positive effect of POHC on work engagement and job crafting during the pandemic.

In synthesizing our arguments, it is reasoned that LHM strengthens the relationship between POHC and work engagement and the indirect effect of POHC on job crafting through work engagement in the context of the pandemic. Therefore, we put forth the following moderation and moderated mediation hypotheses:

**Hypothesis** **3** **(H3).** *LHM moderates the relationship between POHC and employees’ work engagement during the COVID-19 pandemic, such that this relationship is more pronounced when LHM is high than when it is low.*

**Hypothesis** **4** **(H4).** *LHM moderates the indirect effect of POHC on employees’ job crafting through work engagement during the COVID-19 pandemic, such that this effect is more pronounced when LHM is high than when it is low.*

## 3. Method

### 3.1. Sample and Procedure

We gathered survey-based data from employees working in several diverse organizations within South Korea. With the permission of a South Korean online survey company, we had access to a list of employees who were registered on an online survey platform. We contacted 635 full-time employees and sent them a survey invitation, along with an informed consent form. We guaranteed participants’ confidentiality and anonymity to their responses and complied with the ethical standards of the institutional research committee. Once the invited employees agreed to participate, they received an email containing a survey link.

As we aimed to assess the effect of POHC on work engagement and job crafting in the context of the pandemic, we administered surveys after the outbreak of COVID-19 in South Korea (i.e., 20 January 2020). To ensure the temporal precedence of the independent variable over the dependent variable, and reduce common method variance (CMV) [43,44], we collected data at two points in time at one and a half month intervals (i.e., March 2020 (Time 1: T1) and April 2020 (Time 2: T2)). The T1 survey comprised items assessing POHC, leader health mindset, work engagement, and control variables. The T2 survey was designed to measure job crafting. South Korea experienced its first COVID-19 death in February 2020, and the first wave of the pandemic in March 2020—triggered by the surge of confirmed cases in a church in Daegu, South Korea. Thus, our research period was appropriate for assessing the effect of POHC after the onset of the pandemic, since most South Koreans were aware of the risks posed by the pandemic conditions.

Of the 537 employees who participated in the T1 survey, 301 responded to the T2 survey (retention rate = 56.1%). Sixty-three percent of the respondents were women. The average age of the sample was 36.49 (SD = 8.52) years. On average, the participants held their current job for 4.85 (SD = 4.54) years. The respondents were employed in various industries: retail (e.g., departments and supermarkets; 58%), hospitality/tourism (e.g., restaurants, airlines, and hotels; 35%), and banking/insurance (7%). To check for any systematic differences between these industries, we conducted one-way analysis of variance (ANOVA). The results of ANOVA indicate no significant industry differences in terms of POHC (*F* = 0.30, *p* > 0.05), LMH (*F* = 1.44, *p* > 0.05), work engagement (*F* = 0.63, *p* > 0.05), and job crafting (*F* = 1.64, *p* < 0.05).

### 3.2. Measures

In line with prior studies, we used Brislin’s [45] back-translation procedure to construct our surveys. Responses for the variables were made according to a five-point Likert-type scale (1 = strongly disagree, 5 = strongly agree; see Table 1). To assess POHC, we employed the organizational dimension (i.e., four items) of the POHC scale [4]. The leader health mindset was measured using three items from Franke et al.’s scale [35]. Work engagement was assessed using the nine-item scale of Schaufeli et al.’s [46] nine-item scale, which represents vigor, dedication, and absorption. Job crafting was evaluated using Slemp and Vella-Brodrick’s [47] job crafting scale comprising three five-item sub-dimensions (i.e., task, relational, and cognitive crafting). Consistent with prior research on job crafting [48,49], we averaged the scores for the three sub-dimensions to create a single job crafting index.

Age, gender, and job tenure were control variables in our analyses given their potential effects on work engagement and job crafting [50,51,52]. We also controlled for positive and negative affectivity using the Positive and Negative Affect Schedule Short Form [53].

## 4. Results

Table 2 presents the descriptive statistics and the correlations of the variables. As shown in Table 2, all variables exhibited reliability indices greater than 0.70 [54]. We performed a confirmatory factor analysis (CFA) to assess discriminant validity among the variables. The results of the CFA revealed that the proposed six-factor model (i.e., POHC, leader health mindset, work engagement, job crafting, positive affectivity, and negative affectivity) fit the data well in an absolute sense (χ^2^_(608)_ = 1214.30, *p* < 0.05, comparative fit index (CFI) = 0.91, Tucker–Lewis index (TLI) = 0.90, root mean square error of approximation (RMSEA) = 0.06, standardized root mean square residual (SRMR) = 0.06). In addition, all variables displayed acceptable levels of composite reliability (i.e., 0.76–0.94). As reported in Table 2, the average variance-extracted values for the variables were greater than the squared correlation between the focal variable and the others [55]. Considered together, these findings confirm that our measurement scales possess acceptable psychometric properties.

As we collected T1 and T2 data from the same individuals, CMV might have inflated the relationships between the variables. To remedy this problem, we followed the procedures outlined by Podsakoff et al. [56]. We conducted Harman’s single-factor test and found that the proposed measurement model fitted the data significantly better than the one-factor model in which all items loaded onto a single factor (χ^2^_(629)_ = 5254.93, *p* < 0.05, CFI = 0.16, TLI = 0.15, RMSEA = 0.16, SRMR = 0.16; Δχ^2^_(21)_ = 4040.63, *p* < 0.01). Additionally, we estimated an additional latent common method factor (LCMF) on which measurement items proposed in the baseline model were allowed to load (in addition to loading on its respective construct). LCMF accounted for 6.1% of the total variance, which was much smaller than the median method variance (25%) observed in studies using self-reported data [57]. Thus, it is unlikely that our findings were affected by CMV infection.

### Analysis

As analytic tools, we utilized PROCESS Macro 4.0 (Model = 1, 4, and 7) and a bootstrapping procedure (N = 5000) [58]. Hypothesis 1 proposed a positive relationship between POHC and job crafting. We found a significant positive association between these two variables (*b* = 0.15, 95% CI [0.09, 0.22]), supporting Hypothesis 1.

Hypothesis 2 postulated that work engagement mediates the POHC job crafting relationship. As shown in Table 3, the simple mediation analysis demonstrated a significant indirect effect of POHC on job crafting throughout work engagement (*b* = 0.03, 95% CI [0.01, 0.06]). Hence, Hypothesis 2 was supported.

**Figure 1:**
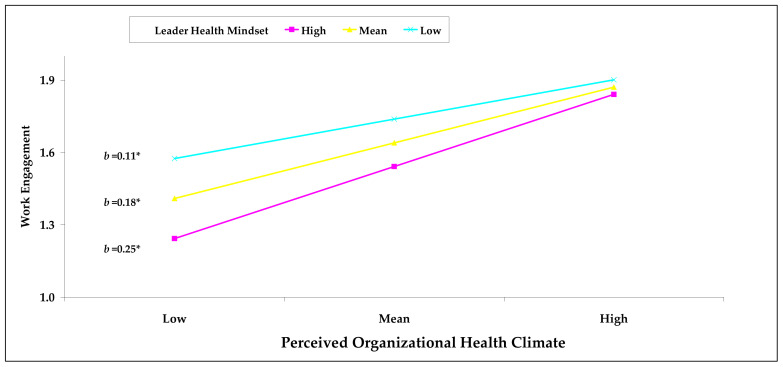
Interaction effect of POHC and LHM on work engagement. Note: N = 301; POHC = perceived organizational health climate; LHM = leader health mindset; *b* = unstandardized coefficient. * *p* < 0.05.

Hypothesis 3 predicted a moderating effect of the leader health mindset on the relationship between POHC and work engagement. Table 4 illustrates that the leader health mindset strengthened the positive relationship between POHC and work engagement (b = 0.07, *p* < 0.01). The results obtained by plotting simple slopes at ±1 SD of the moderator [58] are depicted in Figure 1, and show that the positive association between POHC and work engagement was more pronounced when the leader health mindset was higher than when it was low (high: *b* = 0.25, 95% CI = [0.15, 0.35]; mean: *b* = 0.18, 95% CI = [0.10, 0.26]; low: *b* = 0.11, 95% CI = [0.03, 0.20]). These results support Hypothesis 3.

**Figure 2:**
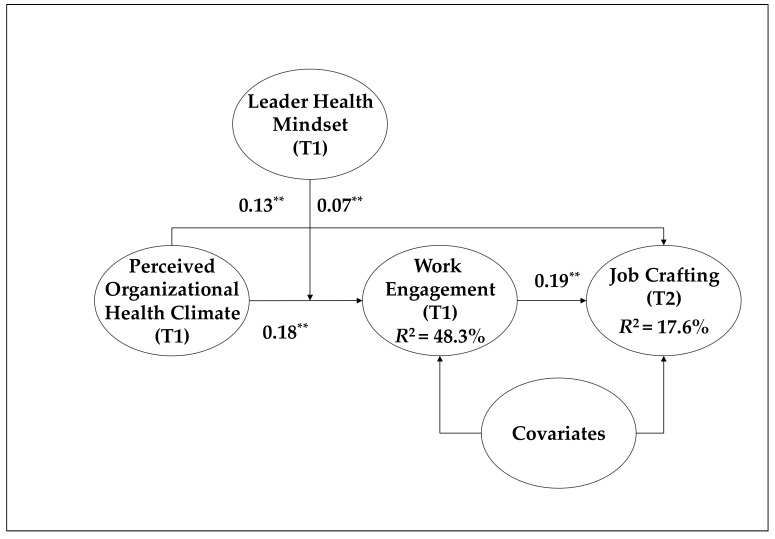
Summary of results; unstandardized coefficients were provided. For model parsimony, the results for the covariates (e.g., gender, age, job tenure, positive affectivity, and negative affectivity) were omitted. ** *p* < 0.01.

Hypothesis 4 posited that a leader health mindset would strengthen the indirect effect of POHC on job crafting through work engagement. As presented in Table 4, the leader health mindset strengthened this indirect effect (b = 0.014, 95% CI = [0.001, 0.033]). Table 5 further demonstrates that the positive indirect effect of POHC on job crafting through work engagement was significant when the leader health mindset was greater than the average (high: *b* = 0.05, 95% CI = [0.01, 0.10]; mean: *b* = 0.04, 95% CI = [0.01, 0.07]). Conversely, when the leader health mindset was low, the positive indirect effect of POHC on job crafting through work engagement became non-significant (low: *b* = 0.02, 95% CI = [−0.00, 0.05]). The results of hypothesis testing are summarized in Figure 2.

## 5. Discussion

Our study aimed to investigate the mediating relationship between POHC, work engagement, and job crafting, and the moderating effect that LHM has on this relationship in the pandemic context. The results of the mediation and moderated mediation analyses supported all hypotheses. As predicted, we found a significant positive relationship between POHC and job crafting. This relationship was significantly mediated by work engagement. LHM exerted a significant moderating effect, and the positive relationship between POHC and work engagement and the indirect effect of POHC on job crafting through work engagement were more pronounced when LHM was high (than when it was low). The theoretical and practical implications of these findings are discussed in the following sections.

### 5.1. Theoretical Implications

The results of our mediation analysis support the indirect effect of POHC on job crafting through work engagement, which corroborates the basic premise of the JD–R model. In this model, work engagement is a key intermediary process underlying the relationship between job resources and work behavior. That is, if organizations provide employees with helpful job resources, their personal resources (i.e., work engagement) increase. Employees mobilize these resources toward core task activities and extra-role behaviors. As such, when employees perceive that their organization has policies and practices that promote employee health, they have greater personal resources. By utilizing these resources, employees can proactively adjust their tasks to their own needs and strengths and improve their relationships with others in the workplace—all of which are crucial behaviors in a pandemic situation. Our findings also support SET by demonstrating the positive relationship between POHC and work engagement and the positive relationship between POHC and job crafting. When employees feel that their organization is genuinely concerned about employee well-being, they reciprocate with positive work attitudes (e.g., work engagement) and behavior (e.g., job crafting) [41]. Our findings present a nuanced approach to the JD–R framework and SET by demonstrating LHM as a boundary condition that fortifies the conducive effect of POHC. JD–R and social exchange theorists have called for more research into the boundary conditions of JD–R and social exchange processes. By presenting LHM as a social resource that fuels the positive impact of POHC, our findings highlight that leaders’ mindsets play a facilitative role in transmitting the positive effect of organizational resources, which implies that employees’ work engagement and job crafting are enhanced when given both organizational and social resources. Likewise, employees are likely to reciprocate with greater work engagement and job crafting when they are treated well by both the organization and supervisor. Overall, these findings suggest that the JD–R model and SET are pertinent theoretical frameworks for elucidating upon the positive relationship between POHC and work outcomes and the moderating effect of LHM during the pandemic.

Our research advances organizational health research by uncovering the interaction effect between POHC and LHM. Kaluza et al.’s [4] findings demonstrate that leaders’ perceptions of organizational health climate were positively associated with their own health mindset. While this study offers insight into the relationship between POHC and LHM, the interplay between POHC and LHM has rarely been explored. This is a critical omission, as both organization and leaders play a significant role in employee work outcomes. Our research fills this research gap by examining the moderation of LHM on the POHC–work engagement–job crafting link. Our research also responds to Klebe et al.’s [1] call for more research into POHC and health-promoting leadership in the pandemic context. To the best of our knowledge, Klebe et al.’s study is the only one that assessed the effectiveness of health-promoting leadership during the COVID-19 pandemic. Taking a further step from their study, which assessed the effect of health-promoting leadership on employee outcomes during the pandemic, our research revealed that POHC had a positive relationship with employees’ work engagement and resultant job crafting, and that this relationship was strengthened by LHM. As such, the present results, coupled with Klebe et al.’s findings, highlight that POHC and LHM are preconditions for motivating employees to remain engaged and proactive during the pandemic.

In studies conducted in non-pandemic settings, health-promoting organizational climate [4] and leadership [26,37,38,39,40,59] exerted positive effects on work outcomes, respectively. In line with these studies, our findings confirmed that POHC and LHM played beneficial roles in employee outcomes during the pandemic, which underscores the importance of POHC and LHM in both pandemic and non-pandemic contexts. However, it should be noted that whether the importance of POHC and LHM increases amid the pandemic remains unknown. As we did not compare the effect of these two variables before and after the onset of the pandemic, we cannot ascertain that the positive effect of POHC and moderating effect of LHM have become more pronounced under the current crisis.

Our research contributes to the job crafting literature by being the first to shed light on the relationship between POHC and job crafting. As noted earlier, prior research that positions organizational climate as an antecedent to job crafting has mainly focused on diversity climate [18] and climate for innovation [19,20] as conducive work contexts for job crafting. As job crafting is a form of proactive behavior that optimizes adaptation to the work environment, an organizational climate that emphasizes flexibility, adaptability, and risk-taking has drawn attention from scholars. However, in a stressful environment such as the pandemic, an organizational climate that promotes employee well-being should be deemed more important than other types of climates [1]. Our research complements prior work on organizational climate and job crafting by demonstrating POHC as a particularly important organizational climate in the pandemic context. Thus, what type of organizational climate is more or less beneficial to job crafting can be better determined by taking environmental conditions into account.

### 5.2. Practical Implications

Considering that the importance of health-promoting climate and leadership increases in turbulent times, such as the COVID-19 pandemic [1], the findings of this study offer valuable insights into how to help employees remain engaged and proactive throughout the pandemic. Our findings, coupled with prior findings denoting a positive association between employees’ subjective perceptions of health climate and their well-being [5], highlight the role of a health-promoting climate in the workplace. To cultivate such a climate, it is critical to facilitate open discussions about health topics within the organization [39]. Open communication and a shared understanding of health issues create shared perceptions of mutual trust and support among top management, middle managers, and employees, which build a positive work climate conducive to employee work outcomes.

Establishing and implementing health-promoting policies and guidelines can be a means of promoting POHC [4]. Organizational health scholars recommend specific methods for creating a health climate (e.g., regular medical check-ups, physical health training programs, mindfulness training) [4]. In addition to these methods, organizations may consider improving working conditions for their employees. Specific examples of such endeavors are using flexible work arrangements, reducing supervisory mistreatment, providing in-house counseling, and offering employees sufficient time for recovery after periods of high workload [26,38]. These practices, when used in combination with organizational health-promoting policies, can contribute to long-term employee well-being by preventing employees from experiencing burnout and stress at work.

In light of our finding that LHM strengthens the beneficial effect of POHC on work outcomes, it is also important to promote LHM to enhance employees’ work engagement and job crafting. As supervisors frequently and closely interact with employees, they should be cognizant of the risks and costs of employee health problems and enhance their sensitivity toward these problems [1,4,59]. Given that leadership competencies are trainable, organizations are advised to train supervisors in health-promoting leadership and mindsets [1,4,37]. Supervisors should understand that employees experience more severe health and stress symptoms resulting from job insecurity, social distancing, and teleworking as a result of the COVID-19-induced restrictions [1]. In this regard, supervisors should pay close attention to employees’ health-related signals during the pandemic. They also need to take proactive actions to protect employees from workplace virus transmission by conforming to COVID-19-related hygiene protocols and guidelines.

### 5.3. Limitations

The findings should be interpreted in light of the following limitations. First, although we employed a two-wave design to reduce CMV, the independent variable and mediator were measured concurrently, precluding the causal inference between these two variables. As engaged employees have positive attitudes toward their organization, they are likely to perceive organizational climate in a positive way. Thus, it is plausible that a high level of work engagement has caused the respondents to evaluate POHC more highly than its actual level. Hence, it is desirable to use three-wave designs to make stronger causal inferences between POHC, work engagement, and job crafting. Moreover, the one-and-half-month interval between the T1 and T2 surveys might be too short a time frame to establish causality between POHC and job crafting. Given that organizational climate exerts a long-term effect on employee outcomes [60], we recommend that future researchers use longitudinal designs that involve a longer timeframe.

Second, notably, we conceptualized and measured the organizational health climate at the individual level. The organizational climate literature differentiates between organizational (i.e., aggregated perceptions of organizational climate) and psychological (i.e., individuals’ perceptions of organizational climate). Consistent with Kaluza et al.’s study [4], we conceptualized organizational health climate as a specific type of psychological climate and labeled it “POHC”. However, the effect of organizational health climate can be better captured when it represents organizational members’ shared perceptions. Therefore, we suggest that future researchers conduct a multilevel study that assesses the effect of POHC shared among organizational members on employee outcomes.

Third, although our findings confirmed the significant effect of POHC on employee work outcomes, we cannot ascertain that health climate is a stronger predictor of such outcomes than other types of organizational climate. As previous studies have revealed that diversity climate and climate for innovation are significant predictors of job crafting [18,19,20], the relative importance of health climate over these climates cannot be determined without the simultaneous examination of the three types of organizational climate. Likewise, the relative importance of LHM over other forms of leader behavior or mindsets in predicting work engagement and job crafting can be more precisely assessed by comparing the effects of different forms of leader behaviors or mindsets in a single study.

Fourth, to enhance the generalizability of our findings, we collected data from the retail, hospitality/tourism, and banking/insurance industries. However, our sample was mostly drawn from the service sector, which might not represent the South Korean population. Thus, the present findings need to be validated in more diverse industries. As the nature of health concerns and issues tend to differ between different types of jobs, employee job type might interact with POHC to affect their work outcomes. Therefore, we recommend that future researchers probe into this possibility in their study of POHC.

Finally, as we conducted our study in the context of the pandemic, COVID-19-related variables need to be included in the research model. It would be a timely and important research topic to explore whether organizational health climate buffers employees against the anxiety and stress brought about by the pandemic. In addition, virtual work arrangements (e.g., telework), which have proliferated since the outbreak of the COVID-19 pandemic, might affect employees’ job crafting. Job crafting researchers have reported that employees are expected to engage in more job crafting under these new work arrangements [61]. Although we did not measure the extent to which employees teleworked in our research, employees’ work mode should be taken into consideration in assessing the effect of POHC on job crafting in the context of the pandemic.

## 6. Conclusions

Our study aimed to explore the effect of POHC and LHM on employees’ work outcomes during the pandemic. The results of our analyses indicate that POHC and LHM synergistically interacted to positively predict work engagement and job crafting. These findings provide a novel understanding of the role of organizational health climate in job crafting by demonstrating, for the first time, that this type of climate acts as an antecedent to job crafting. Furthermore, our findings extend the extant body of research on health-promoting climate and leadership by uncovering the interaction effect of POHC and LHM on work outcomes. Based on our findings, future organizational health research needs to consider both organizational and leadership aspects in assessing the effect of organizational health on work outcomes. Multilevel research investigating the effect of organizational health climate on more diverse outcomes at different levels of organization can expand the insights gained from the present study.

## Figures and Tables

**Table 1:** Measurement items.

Construct	Measurement Items
POHC (T1)	My organization is committed to employee health and well-being.
	My organization provides me with opportunities and resources to be healthy.
	When management learns that something about our work or the workplace is having a bad effect on employee health or well-being, then something is done about it.
	My organization encourages me to speak up about issues and priorities regarding employee health and well-being.
LHM (T1)	My supervisor consciously pays attention to alarming health signals of followers.
	My supervisor realizes when followers arrive at their personal health limits.
	My supervisor notices, in due time, when followers need a break for recovery.
Vigor (T1)	I feel bursting with energy.
	I feel strong and vigorous at my job.
	When I got up this morning, I felt like going to work.
Dedication (T1)	I am enthusiastic about my job.
	My job inspires me.
	I am proud of the work that I do.
Absorption (T1)	I feel happy when I am working intensely.
	I am immersed in my work.
	I get carried away when I am working.
Work Engagement (T1)	Vigor
	Dedication
	Absorption
Task Crafting (T2)	Introduce new approaches to improve your work.
	Change the scope or types of tasks that you complete at work.
	Introduce new work tasks that you think better suit your skills or interests.
	Choose to take on additional tasks at work.
	Give preference to work tasks that suit your skills or interests.
Relational Crafting (T2)	Make an effort to get to know people well at work.
	Organize or attend work-related social functions.
	Organize special events in the workplace (e.g., celebrating a co-worker’s birthday).
	Choose to mentor new employees (officially or unofficially).
	Make friends with people at work who have similar skills or interests.
Cognitive Crafting (T2)	Think about how your job gives your life purpose.
	Remind yourself about the significance your work has for the success of the organization.
	Remind yourself of the importance of your work for the broader community.
	Think about how your work positively impacts your life.
	Reflect on the role your job has for your overall well-being.
Positive Affectivity (T1)	Determined
	Attentive
	Alert
Negative Affectivity (T1)	Afraid
	Nervous
	Upset

Note: Items measured on a scale ranging from 1 (“strongly disagree”) to 5 (“strongly agree”). T1 = time 1; T2 = time 2.

**Table 2:** Means, standard deviations, and correlations for the sample taken.

Variables	M	SD	α	CR	1	2	3	4	5	6	7	8	9
Gender	0.37	0.48	-	-	-								
2. Age	36.49	8.52	-	-	0.15 **	-							
3. Job tenure	4.85	4.54	-	-	0.11 ^†^	0.41 **	-						
4. Positive affectivity	2.65	0.80	0.81	0.82	0.00	0.05	−0.04	**0.60**					
5. Negative affectivity	2.95	0.96	0.87	0.87	−0.11 ^†^	−0.26 **	−0.02	−0.14 *	**0.69**				
6. POHC	2.97	0.94	0.94	0.94	0.04	0.06	0.08	0.08	−0.20 **	**0.78**			
7. LHM	2.98	0.98	0.92	0.92	−0.01	0.06	0.04	0.12 *	−0.20 **	0.60 **	**0.80**		
8. Work engagement	3.06	0.71	0.85	0.92	−0.00	0.21 **	0.15 **	0.60 **	−0.28 **	0.27**	0.18 **	**0.79**	
9. Job crafting	3.30	0.57	0.71	0.76	−0.07	0.07	0.07	0.26 **	−0.01	0.26**	0.13**	0.33 **	**0.52**

Note: N = 301; Gender: 0 = female, 1 = male; POHC = perceived organizational health climate; LHM = leader health mindset; CR = composite reliability; α = Cronbach’ alpha. The bold numbers along the diagonal are the average variance extracted values; ^†^ *p* < 0.10, * *p* < 0.05, ** *p* < 0.01.

**Table 3:** Test of the mediating effect of work engagement on the POHC–job-crafting relationship.

Path	Effect (*b*)	95% CI_low_	95% CI_high_
Total Effect			
POHC → Job crafting	0.15	0.09	0.22
Direct Effect			
POHC → Job crafting	0.13	0.06	0.19
Indirect Effect			
POHC → Work engagement → Job crafting	0.03	0.01	0.06

Note: N = 301; POHC = perceived organizational health climate; *b* = unstandardized coefficient.

**Table 4:** Results for the moderation of LHM on work engagement and job crafting.

Variable	Work Engagement		Job Crafting	
	*b*(se)		*b*(se)	
Gender	−0.08	(0.06)	−0.06	(0.06)
Age	0.01	(0.00)	−0.01	(0.00) ^†^
Job tenure	0.02	(0.01) *	0.01	(0.01)
Positive affectivity	0.50	(0.04) **	0.08	(0.05) ^†^
Negative affectivity	−0.11	(0.03) **	0.05	(0.03)
POHC	0.18	(0.04) **	0.13	(0.03) **
LHM	−0.02	(0.04)		
POHC × LHM	0.07	(0.03) **		
Work engagement			0.19	(0.06) **
*R^2^*	48.3%		17.6%	
Moderated Mediation Index: *b* = 0.014, 95% CI= [0.001, 0.033]				

Note: N = 301; *b* = unstandardized coefficient; se = standard error; Gender: 0 = female, 1 = male; POHC = perceived organizational health climate; LHM = leader health mindset; * *p* < 0.05, ** *p* < 0.01; ^†^ *p* < 0.10.

**Table 5:** Conditional indirect effect of POHC on job crafting through work engagement for levels of LHM.

Moderating Variable	POHC → Work Engagement → Job Crafting		
LHM	*b*	Cl_95%low_	Cl_95%high_
High	0.05	0.01	0.10
Mean	0.04	0.01	0.07
Low	0.02	−0.00	0.05

Note: CI = confidence interval; LHM = leader health mindset; *b* = unstandardized coefficient.

## Data Availability

The data presented in this study are available upon request from the corresponding author.
